# The use of therapeutic untruths by learning disability nursing students

**DOI:** 10.1177/0969733020928130

**Published:** 2020-07-06

**Authors:** Karen McKenzie, Suzanne Taylor, George Murray, Ian James

**Affiliations:** 5995 Northumbria University, UK; 3129 NHS Lothian, UK; 5633 Cumbria, Northumberland, Tyne and Wear (CNTW) NHS Foundation Trust, UK

**Keywords:** Ethics, learning disability, student nurses, therapeutic untruths

## Abstract

**Background:**

The use of therapeutic untruths raises a number of ethical issues, which have begun to be explored to some extent, particularly in dementia care services, where their use has been found to be high. Little is known, however, about their use by health professionals working in learning disability services.

**Research question:**

The study aimed to explore the frequency of use of therapeutic untruths by student learning disability nurses, and by their colleagues; how effective the students perceived them to be as a means of responding to behaviours that challenge; and their level of comfort with using them.

**Research design:**

A correlational design was used to gather data from an online version of the Best Interest Scale, adapted for a learning disability context. Participants were 30 learning disability student nurses (female = 28, ages 18–48 years, M = 26.8, standard deviation = 7.3) studying at a university in the North-East of England.

**Ethical considerations:**

The study was reviewed and received ethical approval from the first author’s university ethics committee.

**Findings:**

Overall, 96% of participants reported using therapeutic untruths. ‘Omission’ was the most frequently used type of therapeutic untruths, the most effective and the type that the students felt most comfortable using. Frequency of use of therapeutic untruths correlated significantly and positively with perceived effectiveness and the level of comfort that the students felt when using them, for all types of therapeutic untruths.

**Conclusion:**

The use of therapeutic untruths by the student nurses was consistent with that found in research in dementia care services in the United Kingdom and abroad. Further research to explore the generalisability of the results to the wider context of learning disability services is needed. The study highlights that there may be a need for more formal guidance and educational input to student nurses in the use of therapeutic untruths with people with a learning disability.

## Introduction

A key aim of nurse education is to train skilled and competent professionals with an appropriate value base. There are differing definitions of professionalism, but common elements are the ability to internalise and work in accordance with the core ethical principles and values of the particular profession and to make decisions that are appropriate to the context, when faced with principles that are in opposition to each other.^1^ Student nurses undergoing this process are shaped by both formal demonstrations of the expected values and standards of behaviour, such as professional guidelines, and informal processes, such as observing how peers and qualified staff behave as part of a `community of practice.^2^

The use of Therapeutic untruths, represents one situation where both student and qualified nurses have to reconcile opposing principles. Therapeutic untruths are a form of deception that are used by staff and others in the best interests of a person who is being supported, for example to reduce distress. These are distinguished from non-therapeutic lies, which instead are used in the interests of the person providing support.^3^ There is a growing body of evidence showing that the use of therapeutic untruths is common in dementia care, with nearly 97% of staff in one study reporting their use^4^ Their use has also been found to occur in services out with the UK.^3^ There has, however, been limited research into the use of therapeutic untruths in learning disability services. In order to address this, the present study aims to explore the experiences of learning disability student nurses in relation to the frequency of use of therapeutic untruths with people with a learning disability, both by themselves and colleagues, how effective they perceive them to be as a means of responding to behaviours that challenge, and the level of comfort of the students with using them.

## Background

The use of therapeutic untruths has raised a number of complex ethical issues and there are conflicting views about their appropriateness and acceptability. On the one hand, therapeutic untruths are seen as unprofessional, immoral, a fundamental betrayal of trust, or a form of abuse by those who oppose their use,^5,6^ although people with dementia themselves condone their use under certain circumstances, that together constitute an action that is in the best interests of the person.^6,7^ On the other hand, therapeutic untruths are seen as a means of communication with people who have memory loss, a decline in functional ability and who may create their own reality,^8^ or as a strategy to alleviate anxiety.^9^ Indeed, it has been suggested that the ethical principal of beneficence and preventing harm to others may well be interpreted in some situations as requiring the professional to use therapeutic untruths.^9^ Healthcare professionals, therefore, face a dilemma in relation to the use of therapeutic untruths and must consider the legal, ethical and clinical issues when making decisions about this practice.^6,10^ This means that the extent to which therapeutic untruths are used may be influenced by the ethical stance that staff adopt in relation to therapeutic untruths and the associated moral discomfort if they use them in practice.

More recently, guidance has been published regarding the care of older adults, to help clarify the factors that influence whether the use of therapeutic untruths would be appropriate for a given individual or not, with the key focus being on the ‘best interests’ of the person.^11,12^ This guidance has been developed in the context of previous research in dementia care services, and while the principles are generalisable to other potentially vulnerable groups, such as people with a learning disability, there has been no research that has explored the use of therapeutic untruths with professionals working in this field.

All people with a learning disability have lifelong and significant difficulties with their cognitive and adaptive skills,^13^ but comprise a heterogeneous group, with their difficulties ranging from mild to profound. For some, this means they may have problems communicating their needs and wishes and may use behaviours that challenge, such as aggression, to express that their needs are not being met appropriately.^14^ It may, therefore, be that another factor that influences the use of therapeutic untruths in learning disability services is the extent to which staff view them as an effective strategy, in the best interests of the person, for managing behaviours that challenge.

In order to address the dearth of research in this area, the present study explores the experiences of learning disability student nurses in relation to the frequency of use of therapeutic untruths with people with a learning disability, both by themselves and colleagues; how effective they perceive them to be as a means of responding to behaviours that challenge; and the level of comfort of the students with using them.

## Research design

### Method

The study used a correlational design, with the data being collected via an online questionnaire.

Therapeutic untruths were measured by the Best Interest Scale,^4^ and was originally developed for use in dementia services. The use of the measure in the present study allows some comparisons to be made across different specialism. The measure was based on Blum's classifications of types of deception^15^ and is in three parts. The first section presents three scenarios, the responses to which are coded in terms of the presence of different types of Therapeutic untruths (omission, going along, white lie, outright untruth) or no Therapeutic untruths. These scenarios were adapted to make them more relevant to working with people with a learning disability (see Table 1). A description and example of each type of Therapeutic untruths, sample responses from participants, where available, and codes are provided in Table 1. If a scenario had a response which was coded as containing a Therapeutic untruths it was given a score of 1. These were added across the three scenarios to provide a total Therapeutic untruths scenario score, with a range of 0–3.

**Table 1.** Adapted scenarios with sample coding of responses.

Types of therapeutic untruths				
	Omission *Failing to provide the person with the complete information to hand with the intention of reducing distress or getting them to do something.* *Not telling whole truth.* *Example: Not telling a person that a loved one is seriously ill*	Going along *Failing to correct a person’s misperceptions of a situation, which were due to their confusion, misunderstanding, hallucination or unusual thought processes.* *Example: Responding ‘That’s nice’ when the person says a deceased family member will be visiting that day.*	White Lie *An untruth, which is perceived to be a minor lie because ‘qualifications’ are used. Furthermore, the actual message may be correct at some time in future.* *Example: Saying ‘I think [favourite staff member] will be here soon.*	Outright untruth *Information that is completely untrue, and there is no likelihood the event will come true.* *Example: ‘You will have to leave your home if you do that once more’.*
Scenario 1: John has a mild intellectual disability and becomes upset if his routine changes unexpectedly. His favourite staff member was due to start work 5 min ago, but has called in sick. John is beginning to become agitated.	‘Reassure John that there are other members of staff to support him’.	No examples available.	No examples available.	Tell the patient that the member of staff has gone to do something for the patient that involves the patient’s interests but agree this with the member of staff first.
Scenario 2: Amy’s mother is terminally ill in hospital and has been unable to visit her as a result. Amy is not aware that her mother is dying and says to you ‘I’m sure mum will come to see me today’.	Explain to Amy that her Mum is unable to come today.	I’m sure she is looking forward to seeing you.	I would say that it’s possible that Amy’s mum might come but she may have other things to do and might not be able to come and visit today.	I’d say you’re unsure of her plans but I’m sure she will come visit when she is free to try occupy them.
Scenario 3: Alex has a favourite t-shirt and becomes aggressive if asked to wear anything else. The original shirt had to be thrown out because it was damaged by the washing machine. His mother has bought a very similar t-shirt to replace it. When you offer this to Alex in the morning, he looks at it for a long time and asks, ‘Is this my favourite t-shirt?’	I’d say it certainly looks like it.	No examples available.	Say I think it is.	I would probably lie and say yes, the washing machine made it ‘really’ clean.

The second section provided a description and examples of the different types of Therapeutic untruths, in order to provide participants with the information they required for the following section. The third section presented these Therapeutic untruths to participants, who were asked to rate how frequently they used, and had observed colleagues using, each type. The latter related to observing qualified nurses and other staff while the students were on placement. Responses were on a 5-point scale from 4 (often) to 0 (never). The participants were then asked to rate their perception of the effectiveness of each type of Therapeutic untruths as a method of successfully managing behaviours that challenge and, finally, their level of comfort using each type. These ratings were on a 5-point scale from 4 (extremely) to 0 (not at all).

### Participants

Thirty learning disability student nurses took part, of whom 8 were in their first year of training, 6 their second year and 16 in their third year. All but two were female and described themselves as British or White British (n = 28, 93.3%). Ages ranged from 18 to 48 years (M = 26.8, standard deviation (SD) = 7.3). Participants were eligible to take part if they were aged 18 years or older and were a nursing student working with people with a learning disability.

### Procedure

Participants were recruited from the group of students undergoing learning disability nursing training at one university in the North East of England. All potential participants (n = 63) were emailed information about the study and the link to the online questionnaire. On accessing the survey, the participants received more detailed information to ensure they had sufficient information to provide informed consent. They recorded consent by clicking on a button that indicated that they agreed to participate, before completing the measures outlined above. They also provided basic demographic information. All responses were anonymous.

### Ethical considerations

Ethical approval for the study was granted by the first author's institutional ethics committee (reference number: 13910). The study was exploring a topic that raises a number of potentially conflicting ethical considerations: the use of untruths in the best interests of a person with a learning disability. To try to minimise the likelihood of the student responses being influenced by social desirability factors, the study was conducted online, rather than face to face and responses were completely anonymous, as participants generated their own code (for the purpose of withdrawing their data should they wish to do so at a later stage). Participants were given the contact information of the researchers if they had any questions or concerns following completion of the study. Participation was entirely voluntary and participants were advised that they could miss out any questions that caused them discomfort and stop at any time.

### Materials

Therapeutic untruths were measured by the Best Interest Scale,^4^ which was based on Blum’s classifications of types of deception^15^ and was originally developed for use in dementia services. The measure is in three parts. The first section presents three scenarios, the responses to which are coded in terms of the presence of different types of therapeutic untruths (omission, going along, white lie, outright untruth) or no therapeutic untruths. These scenarios were adapted to make them more relevant to working with people with a learning disability (see Table 1). A description and example of each type of therapeutic untruths, sample responses from participants, where available, and codes are provided in Table 1. If a scenario had a response which was coded as containing a therapeutic untruth, it was given a score of 1. These were added across the three scenarios to provide a total therapeutic untruth scenario score, with a range of 0–3.

The second section provided a description and examples of the different types of therapeutic untruths, in order to provide participants with the information they required for the following section. The third section presented these therapeutic untruths to participants, who were asked to rate how frequently they used, and had observed colleagues using, each type. The latter related to observing qualified nurses and other staff while the students were on placement. Responses were on a 5-point scale from 4 (often) to 0 (never). The participants were then asked to rate their perception of the effectiveness of each type of therapeutic untruths as a method of successfully managing behaviours that challenge and, finally, their level of comfort using each type. These ratings were on a 5-point scale from 4 (extremely) to 0 (not at all).

### Procedure

Participants were recruited from the group of students undergoing learning disability nursing training at one university in the North-East of England. All potential participants (n = 63) were emailed information about the study and the link to the online questionnaire. On accessing the survey, the participants received more detailed information to ensure they had sufficient information to provide informed consent. They recorded consent by clicking on a button that indicated that they agreed to participate, before completing the measures outlined above. They also provided basic demographic information. All responses were anonymous.

## Results

### Therapeutic untruths used in scenarios

The most common therapeutic untruths used in scenario 1 was ‘omission’ (n = 14), with the same number giving a response that indicated no therapeutic untruths. One person used an ‘outright untruth’. Omission was again the most common therapeutic untruth in scenario 2 (n = 12), with five people not using a therapeutic untruth, three ‘going along’, eight telling a ‘white lie’ and one using an ‘outright untruth’. In scenario 3, the most common response was an ‘outright untruth’ (n = 10), followed by no therapeutic untruth (n = 9), ‘omission’ (n = 8) and ‘white lie’ (n = 2). The range of therapeutic untruths across the scenarios was 0–3, with a mean of 2.0 (SD = .87).

### Use of therapeutic untruths by self and colleagues

All but one of the participants reported using at least one type of therapeutic untruth, at least occasionally, and all reported observing their colleagues using some form of therapeutic untruths. Table 2 provides information about the reported frequency with which therapeutic untruths were used by the students and observed by them being used by their colleagues. Table 3 provides information about perceived effectiveness and comfort in using each type of therapeutic untruth.

**Table 2.** Number and percentage for reported frequency of use for each type of Therapeutic untruths by self and colleagues.

Type of Therapeutic untruths	Self No. and %					Colleagues No. and %				
	Never	Rarely	Occasionally	Quite often	Often	Never	Rarely	Occasionally	Quite often	Often
Omission	1 (4%)	6 (24%)	10 (40%)	5 (20%)	3 (10%)	0 (0%)	3 (11.5%)	10 (38.5%)	9 (34.6%)	4 (15.4%)
Going along	1 (4%)	10 (40%)	6 (24%)	8 (32%)	0 (0%)	0 (0%)	4 (15.4%)	12 (46.2%)	7 (26.9%)	3 (11.5%)
White lie	3 (12%)	7 (28%)	3 (12%)	9 (36%)	3 (12%)	3 (11.5%)	2 (7.7%)	7 (26.9%)	11 (42.3%)	3 (11.5%)
Outright lie	17 (68%)	5 (20%)	2 (8%)	1 (4%)	0 (0%)	12 (46.2%)	9 (34.6%)	3 (11.5%)	2 (7.7%)	0 (0%)

TU: therapeutic untruth. Total number of participants providing responses varied between 24 and 26 for the different questions.

**Table 3.** Number and percentage for reported effectiveness and comfort using each type of Therapeutic untruths.

Type of Therapeutic untruthss	Perceived effectiveness No. and %					Level of comfort using No. and %				
	Not at all	Slightly	Moderately	Very	Extremely	Not at all	Slightly	Moderately	Very	Extremely
Omission	0 (0%)	4 (15.4%)	13 (50%)	3 (11.5%)	6 (23.1%)	2 (7.7%)	6 (23.1%)	10 (38.5%)	6 (23.1%)	2 (7.7%)
Going along	3 (12%)	4 (16%)	9 (36%)	7 (28%)	2 (8%)	4 (16%)	4 (16%)	14 (56%)	3 (12%)	0 (0%)
White lie	0 (0%)	10 (40%)	4 (16%)	11 (44%)	0 (0%)	3 (12%)	10 (40%)	4 (16%)	8 (32%)	0 (0%)
Outright lie	11 (44%)	8 (32%)	6 (24%)	0 (0%)	0 (0%)	17 (70.8%)	5 (20.8%)	2 (8.3%)	0 (0%)	0 (0%)

TU: therapeutic untruth. Total number of participants providing responses varied between 24 and 26 for the different questions.

### Relationship between use of therapeutic untruths, perceived effectiveness and level of comfort using them

Table 4 illustrates the Spearman’s correlation between the frequency of use of therapeutic untruths by participants, how effective they perceive them to be as a means of responding to behaviours that challenge and their level of comfort with using them. Table 4 shows positive, significant relationships between the frequency of use of all types of therapeutic untruths and both the extent to which they are perceived as effective and the participants’ level of comfort in using them. As level of comfort and perceived effectiveness increase, so does the frequency of use and vice versa.

**Table 4.** Correlation between the frequency of use of Therapeutic untruths, perceived effectiveness and level of comfort with using them.

Type of Therapeutic untruths	Perceived effectiveness of Therapeutic untruths	Reported level of comfort using type of Therapeutic untruths
Omission	.522**	.468**
Going along	.498**	.585**
White lie	.459*	.413*
Outright untruth	.690**	.724**

TU: therapeutic untruth.

*p < 0.05 level (two-tailed).

**p < 0.01 level (two-tailed).

## Discussion

To our knowledge, this study is the first to explore the reported and observed use of therapeutic untruths by health staff with people with a learning disability. We found that all but one of the student nurses (96%) reported using some form of therapeutic untruths at some time during their training and all reported observing their colleagues doing so. In addition, only one student did not give any response that was a therapeutic untruth to the three scenarios. These figures are consistent with the levels of use of therapeutic untruths found in dementia care services^4^ and suggest that the use of therapeutic untruths may be as common in learning disability services as it has found to be in dementia care services. The therapeutic untruth that was reported as being used most commonly by the students and observed in their colleagues was ‘omission’, with ‘outright untruths’ being the least commonly used. This pattern was observed in responses to the scenarios, with the exception of scenario.^3^ Here, the most common response was an ‘outright untruth’.

Our results also found significant positive correlations between the reported frequency of use of the different types of therapeutic untruths and perceived effectiveness and comfort using them. This indicates that the more the student perceived a therapeutic untruth to be an effective response to behaviour that challenged and the more comfortable they felt using the therapeutic untruths, the more frequently they were likely to use it. There was some variation in both level of comfort of using different types of therapeutic untruths and their perceived effectiveness, with ‘omission’ being seen as the most effective and comfortable to use, while ‘outright untruths’ were the least in terms of both variables. This discomfort with ‘outright untruths’ may stem from moral judgements about the acceptability of particular types of untruths, in the context of guidance that health professionals should be honest in their interactions with those they care for.^16^

The results may also reflect the fact that the students were asked about the use of therapeutic untruths in the context of effectiveness as a strategy for managing behaviours that challenge. ‘Omission’ may have been viewed by participants as a form of evasion, which has been identified as a reactive strategy that can be used appropriately as part of a positive behavioural approach to behaviours that challenge.^17^ Research in dementia care services has also found that the use of therapeutic untruths is viewed as more acceptable in contexts where the person or others are considered to be at risk of harm,^18^ or in response to potential or actual aggression.^3^ Our results suggest that the use of therapeutic untruths may be influenced by judgements about which response may be most appropriate in a given circumstance, as well as considerations of how much of a deception a particular therapeutic untruth represents, although the correlational design of the study means that a causal relationship between effectiveness, level of comfort and use of therapeutic untruths cannot be assumed.

It should also be noted that we did not ask the students directly about the ethical acceptability of the use of each type of therapeutic untruth. Instead, their reported level of comfort was used as an indication of this. It may be, however, that the students interpreted the question about their level of comfort with using particular therapeutic untruth in the context of how effective they felt they were as strategies for responding to behaviours that challenge, rather than how ethically acceptable they considered them to be. It is evident from reports of the abuse of some people with a learning disability that not all practices that may be considered by staff as effective in managing behaviours that challenge would also be considered to be ethically acceptable.^19^ Further qualitative research may be helpful in exploring the relationships between use of therapeutic untruths, perceived effectiveness, level of comfort and ethical acceptability.

There was a reported high frequency of use of therapeutic untruths by the students and of observing their use by colleagues. The latter would have included both qualified nurses and other staff observed while the students were on placement. The use of therapeutic untruths, as modelled by qualified nurses and other staff, may have influenced their use by the student nurses, for example, the students integrating these approaches as part of what they considered to be the informal processes of the nursing community of practice.^2^ This highlights a need to develop or adapt guidance on the use of therapeutic untruths with people with a learning disability, such as that outlined by the Mental Health Foundation,^12^ and to address the topic of using therapeutic untruths in student nurse education and in the continuing professional development of qualified staff. This may help to ensure that both qualified and student nurses are given more formal support in their decision making about the use of therapeutic untruths. Research^20^ to help prepare student nurses to work in dementia care services found that a practical workshop that covered the use of therapeutic untruths as part of an overall communication strategy resulted in the students feeling more competent and accepting of the use of therapeutic untruths. A workshop with qualified clinicians led to a greater awareness of their own use of therapeutic untruths and of the training and supervision needs of staff in their use.^8^ Similar approaches may be helpful for those working in learning disability services.

The research had a number of limitations. The sample size was relatively small, the participants were students studying at only one university and they were predominantly female. This limits the extent to which the results can be generalised and highlights the need for further research with larger and more diverse samples. In addition, while the types of therapeutic untruths were based on previous work,^15^ and have been used to assess the frequency of use of therapeutic untruths in those working in dementia care services,^4^ and as such appear to have face validity, we were unable to find research exploring their other psychometric properties and did not validate the measure ourselves. This is an important area for future research.

## Conclusion

We found that the student learning disability nurses reported using and observing the use of therapeutic untruths by colleagues at a level consistent with that found in research in dementia care services. Our research highlights that there may be a need for more formal guidance and education for staff working in learning disability services about the use of therapeutic untruths to ensure that their use is consistent, in the best interests of those being supported and in the context of a wider positive behavioural support approach to addressing behaviours that challenge. There is also a need for further research to identify which factors may influence their use in learning disability services and to obtain the views of those being supported about the circumstances under which the use of therapeutic untruths might be considered acceptable.
